# ‘All About’ Extremophiles

**DOI:** 10.12703/r/12-27

**Published:** 2023-11-07

**Authors:** James A Coker

**Affiliations:** 1 Center for Biotechnology Education, Advanced Academic Programs, Krieger School of Arts and Sciences, Johns Hopkins University, 3400 N. Charles St., Baltimore, MD 21218, USA

**Keywords:** Biotechnology, disease, astrobiology, replication, repair, transcription, genomics, proteomics, transcriptomics, extremophiles, acidophile, alkaliphile, halophile, piezophile, psychrophile, radiophile, thermophile, mesophile

## Abstract

Despite common perception, most of Earth is what is often referred to as an ‘extreme environment.’ Yet to the organisms that call these places home, it is simply that (home). They have adapted to thrive in these environments and, in the process, have evolved many unique adaptations at the molecular- and ‘omic-level. Scientists’ interest in these organisms has typically been in how they and their products can be harnessed for biotechnological applications and the environments where they are found, while the general public’s veers more toward a fascination with their deviation from the ‘norm’. However, these organisms have so much more to tell us about Life and the myriad ways there are to perform ‘simple’ biological processes.

## Introduction

In our human-centric view of the planet Earth, we tend to think of ourselves as being in the ‘Goldilocks zone’^1^ - not too hot or cold, protected from radiation, and filled with all the things necessary for Life to exist. To some extent, this is true; however, this view keeps us from acknowledging several basic facts, including that the Earth is mostly a cold place (over 90% of its oceans are at or below 5°C^2^, and it has an average temperature of around 15°C), and several conditions we humans consider ‘normal’ (i.e., 20% oxygen in the air) actually make us extremophiles from the point-of-view of other species.

This last point can be said for a variety and plethora of environments, including those that are acidic, alkaline, and/or saline. In each of these ecosystems, like in all others, the organisms present are subjected and respond to survival pressures. Thus, these organisms are merely the product of evolution and the best adapted to their environment. So, from that perspective, there is nothing extreme about them, and we (humans) are the extremophiles.

The attention given to the adaptive strategies ‘extremophilic’ organisms have evolved emphasizes their ‘extremeness’ (i.e., how they adapted to thrive in places where humans would find it difficult to survive) instead of looking at their abilities/characteristics like other organisms. So, instead of yet another review cataloging these traits, I would like to focus here on as many aspects as possible regarding these organisms. Those that have been previously covered in extensive detail (e.g., cellular adaptations^3–7^ and biotech applications^6–9^) will be given less time/space compared to others like ‘omics, the information transfer systems, and disease.

One final word before I begin; I know that having ‘all about’ in a title inevitably means I will miss a topic and have set an impossible bar to reach (and possibly anger one or two folks as a result). However, I hope the title and this review serve as a reminder or a statement that extremophiles are more than just ‘weird’ organisms surviving in ‘strange’, hard-to-reach places that invoke the occasional voyeurism from the rest of the scientific community and public at large. They are wonderfully complex organisms that teach us where the ‘edges’ of Life are, if such a thing even exists, and the myriad ways there are to do ‘simple’ biological processes in the Universe. And, if I may be so bold, they are the best examples of the variety Life has to offer.

## Cellular Adaptations

### pH – Acidophiles and Alkaliphiles

With few exceptions, these organisms maintain a cellular pH near neutral. Therefore, their major cellular adaptations are mechanisms that regulate the hydrogen ion (H^+^) concentration inside the cell. Acidophiles accomplish this by having evolved the following: cell membranes that are more impermeable to protons (compared to other organisms); reduced pore size in membrane channels; net positive potential charge inside the cell; and proton pumps that actively pump protons out of the cell. Alkaliphiles have evolved the following: negatively charged cell wall; acidic secondary cell wall; employment of sodium and potassium antiporters; and production of acids^10–12^.

### Halophiles

Individual organisms have evolved one of two strategies, often referred to as salt-out and salt-in. Organisms that adopt the salt-out approach have evolved to ‘keep the world out’, like acidophiles and alkaliphiles, which results in differing concentrations of ions inside compared to outside the cell. However, in contrast to pumping ions out of the cell, these organisms produce and accumulate large quantities of organic osmolytes to achieve a rough parity in concentration on both sides of the external membrane. This comes at quite an energy cost to the cell; however, for some, the molecules can be recycled and used as an energy source once the salinity lowers^13,14^.

The organisms that adopt the salt-in approach have molar concentrations of ions inside their cell membrane and typically live in environments where salinity does not change dramatically^15^. As such, the adaptations required to live/thrive require a different scale of evolutionary change (compared to salt-out) for these organisms to have functional biochemical processes in what is often a cellular milieu containing three (or higher) molar concentrations of ions. These include a proteome where proteins are covered in acidic residues where the negative charges coordinate water molecules on their surface to keep them hydrated and resist precipitation^16,17^.

### Piezophiles

Initial studies of these organisms were either inconclusive or did not show any adaptations to growth under high hydrostatic pressure. However, more recently, studies have begun to show mechanisms of adaptation^18^. These include genome modifications and alterations in gene expression as well as more subtle changes such as the modification of proteins (flexibility and compressibility)^19^, and activation of stress response systems^20^. These studies have led to the discovery of co-solutes (b-hydroxybutarate and a b-hydroxybutarate oligomer) that accumulate in the cell to counteract the effects of pressure^21^.

### Radiophiles

The effects on survival of two types of radiation (ionizing/gamma and UV) have been studied. UV radiation is often the more subtle of the two in terms of damage, typically resulting in thymine dimers and 6-4-photo products, compared to double-stranded breaks. For UV damage repair, extremophiles use the well-studied mechanisms of photoactivation, base and nucleotide excision repair, and homologous recombination. However, they also employ pigments (carotenoids), dismutases, and hydroperoxidases as a form of photoprotection^22^.

Ionizing radiation, on the other hand, has been shown to be more damaging as it affects not only the nucleic acids in a cell but also the proteins and lipids and induces persistent oxidative stress - a toxic combination for most organisms. To counter these, radiophiles use the well-known processes of base and nucleotide excision repair and homologous recombination; however, these have evolved to be faster and more accurate than in their more radiation-sensitive counterparts. To counteract damage to proteins and lipids, which are sometimes lethal before double-stranded breaks form, these organisms evolved mechanisms that eliminate oxidized macromolecules, protect proteins from damage, and suppress reactive oxygen species. Many radiophiles also have multiple copies of their genome as well as a more condensed nucleoid, which is thought to increase the efficiency of DNA repair and limit the spread of the affected nucleic acids^23,24^.

### Temperature – Psychrophiles and Thermophiles

As with pressure and radiation, cells/organisms cannot keep temperature ‘out’ of the cell, so they must adapt mechanisms to thrive under extremes. Adaptations to temperature have been demonstrated to involve both well-known stress response mechanisms as well as finer adjustments such as altering the type and quantity of specific amino acids, adjusting the flexibility of proteins, producing antifreeze and cold/heat shock proteins, and outer membrane modifications^25,26^.

## ‘Omics and Systems Level Adaptations

In addition to the cellular adaptations, it is becoming clear that extremophilic organisms have also adapted their genomes – namely its size and composition, proteomes, transcriptomes, etc. – to adapt to specific niches^4^. In terms of genome size, studies have shown that acidophiles are smaller in comparison to alkaliphiles, and thermophiles are smaller compared to mesophiles and psychrophiles^4,27^. This reduction in size (streamlining) is a well-reported adaptation to life in stressful environments^28^. For radiophiles, the ability to reassemble the genome in a short time frame is key to surviving the lethal number of double-strand breaks and mutations induced by periods of intense exposure^24^. In terms of genome composition, the instability of nucleic acids at high temperatures is a serious issue for (hyper)thermophiles. To remain alive, they have evolved several genome-wide adaptations, including a high G+C content in the stems of tRNA and rRNA, stabilizing structures via small ligands (Na^+^, K^+^, Mg^2+^), and covalent modification of nucleosides^29^. Psychrophiles have reduced the number of aspartic acid, glutamic acid, proline, and arginine residues in specific groups of proteins (e.g., DNA metabolism and transcription)^25^. Insertion sequences have also been widely suggested to be important in evolutionary adaptations^30,31^; however, despite their prevalence in archaeal and bacterial genomes, very few have been shown to be important for life at extremes^32^.

For proteomes, when looking at pI values, acidophiles and alkaliphiles have a bimodal distribution (one acid and one alkaline peak). However, acidophiles have roughly the same number of acidic and alkaline proteins, while alkaliphiles have significantly more acidic proteins compared to alkaline^33^. In halophiles, organisms that adopt the salt-in adaptation show a strong skew toward an acidic proteome with very few alkaline proteins^34^. Proteomes of psychrophiles show amino acid substitutions that result in greater protein flexibility^25^.

For transcriptomes, piezophiles show an upregulation of genes encoding proteins involved in cell motility, cell wall/membrane biogenesis, extracellular structures, and toxin-antitoxin response when compared to non-pressure-sensitive species of the same genus^35^. One type of polyextremophile (salinity and low temperature) shows other specific responses, including the upregulation of gas vesicles, cold-specific general transcription factors, and gene regulators^36,37^.

## Information transfer

### Replication/Repair

Like their mesophilic counterparts, extremophiles contain the well-studied replication machinery that is Domain-specific - one origin of replication recognized by a DnaA homolog in Bacteria and *orc*1/*cdc*6 family members that bind to either one or multiple origins of replication in Archaea and Eukarya. The process of replication in both archaeal and eukaryal cells is quite similar; however, there is evidence that certain origin-of-replication proteins are essential for specific replication origin sites in archaeal genomes, suggesting archaeal genome replication may be more regulated than their eukaryal counterparts^38^.

Faithful DNA replication is dependent on efficient repair of DNA, and DNA damage due to extreme conditions is a common reason for the death of mesophiles. As such, most extremophiles have evolved the known repair systems to function in *in situ* conditions^39^. For thermophiles and radiophiles, repair plays an outsized role in survival as these niches result in higher amounts of DNA damage. Since the damage is similar in both environments^40^, it has been hypothesized that properties that allow hyperthermophiles to thrive in their environments also allow them to withstand high levels of radiation^23^. Other elements of DNA repair, like RecG, have also been shown to be important^41^.

### Transcription

In terms of the components of the pre-initiation complex and mechanisms of action, extremophiles use and operate in a manner that is specific to an individual organism’s Domain of Life (i.e., sigma factors for Bacteria; TBP, TFB, TFE, and TFS for Archaea; and TFIIB, TFIID, TFIIE, etc. for Eukarya). However, there is one notable exception found within the Archaea, where biochemical, genetic, genomic, and transcriptomic studies have shown that they use specific pairings of their basal transcription factors (TBP and TFB) to regulate gene expression^36,42–45^. For example, a TbpD-TfbA complex will regulate a certain group of genes, while a TbpC-TfbG complex regulates a different set. Interestingly, a similar mechanism of pairing of basal transcription factors has also been documented in the metazoans^46–48^.

When looking at transcription regulators, however, the image of the archaeal machinery changes to something more akin to bacteria. Transcriptional regulators in archaeal cells are from the prokaryotic families ArsR/SmtB, Lrp/AsnC, MarR, and TrmB^49,50^. However, studies have shown that archaeal transcription factors have differentiated from their bacterial counterparts, including the following: being smaller compared to the average of other proteins in the cell, predominantly using the helix-turn-helix motif in the DNA-binding domain, encoding fewer (based on whole-genome studies), using transcription factors that are uncommon (compared to bacterial cells), and being mostly single-component^50^.

## Biotech applications and concerns

For the applications of enzymes/products of extremophiles, there have been five major success stories: biofuels (increased efficiency of production using enzymes from thermophiles)^51^; biomining (increased capture of minerals/ores with acidophiles and thermophiles)^52^; carotenoid production (production by halophiles)^16^; detergents (addition of lipases/proteases from psychrophiles, alkaliphiles, and thermophiles)^53^; and PCR automation (via use of thermostable polymerases from thermophiles)^54^. In addition to these, a plethora of possibilities have been suggested, including generating bioplastics (from polyhydroxyalkanoates)^55^, lactose-free dairy products (from cold-active beta-galactosidases)^56^, high-fructose corn syrup (from starch-hydrolyzing enzymes)^57^, and vaccines (from gas vesicles of halophiles)^58^.

In addition to the typical look at solutions/successes offered by extremophiles, it is also important to look at the ‘problems’ they cause as well. A few examples are – spoiling milk and other dairy products (due to proteolytic activity of psychrophiles)^59,60^, contaminating dairy powders (due to the presence of the spore form of thermophiles)^61^, spoiling fruit juice (due to the presence of acidophiles like *Alicyclobacillus* species)^62^, spoiling salted fish (due to the presence of halophiles)^63^, and corroding oil pipelines (due to the activity of thermophiles)^64,65^.

## Disease

As the years have gone by and our understanding of the human body and its contents, as well as what is an ‘extremophile’ have changed, so has the nature of this topic. Gone are the days when the human body was thought of as being composed of only eukaryotic ‘human’ cells, and all others are just ‘passing through.’ The research on the human microbiome has shown us that each person has a higher number of microbes than human cells. But maybe more importantly, there are roughly 100x more microbial genes compared to human genes in any individual of *Homo sapiens*^66^. As such, we must shun any naïve ideas that the microbes that cover and are found throughout our bodies do not play a role in most/all processes – including disease.

In addition to the above-mentioned problems (i.e., contamination and spoilage of beverages and food) causing disease in humans, there is at least one example of an extremophile being the root cause of disease in mammals - the acidophile *Coxiella burnetii*, which causes Q fever^67^. Additionally, there are many examples where the root cause and consequence are less clear due to the intrinsic nature of human-microbe interactions across the gut (microbiota)-brain axis^68^. These interactions are well documented and have shown that the gut microbiome helps to maintain brain chemistry and cognition while the nervous system modulates the GI tract in multiple ways, thereby sustaining the gut microbiome^69,70^. As such, changes in the gut microbiota impair neural homeostasis, which results in neural and chemical changes^70,71^. Multiple studies have shown the differences in the gut microbiota between healthy and diseased individuals. The Archaea and extremophiles often get overlooked in these studies. However, there are some that show quite remarkable findings, such as a higher abundance of halophilic archaea in patients with colorectal cancer (e.g., *Halopelagius, Halorubrum, Halococcus*) and adenoma (e.g., *Halohasta, Haloferax,* and *Natrinema*)^72,73^. Several archaeal metabolites (including halophilic and thermophilic species) have potential links to cancer^74^, including short-chain fatty acids, polyamines, indoles, and acetaldehyde^75,76^. Finally, it is important to remember that bacteria and archaea have been reported in all parts of the body^77^. As such, it is difficult to exclude them from the list of causative agents of disease.

## Astrobiology and the Origin of Life on Earth

As we look at the extremes where life can exist on our planet: well below freezing to above 120°C; across the entire pH scale; beyond saturating conditions of NaCl and KCl; and UV/gamma radiation levels 10s to 100s of times above what humans can survive, it is natural to wonder if these organisms can give us insight to what we have hypothesized was the chaotic origins of our planet and what the limits of Life are (if limits exist) across the Universe.

Testing the ability of living organisms to survive in space and low Earth orbit has been an ongoing exercise for just about as long as there have been space programs. These started in the 1960s and have continued, often as a part of larger experimental programs like Bion/Kosmos^78^, BIOPAN^79^, EXPOSE^80^, O/OREOS^81^, and Tanpopo^82^. These endeavors started with a ‘simple’ goal of looking for extraterrestrial life; however, they have grown into experiments to test the limits of life and determine if there is any underlying validity to theories like panspermia. Throughout, the study of extremophiles has been at the center of these undertakings. It has been documented that species of radiophiles can survive up to 48 years in space^83^, and even the seeds of plants can produce viable offspring after one and a half years of exposure in space^84^. These longevity-of-survival types of studies eventually led to habitability studies where overlays were made of the theorized environments on known exoplanets and the known extremes of life on Earth^85,86^. Conclusions from these studies have shown that there is a high probability for environments that can support life on exoplanets and their terrestrial moons.
